# Chronic heart failure management in primary healthcare in Poland: Results of a nationwide cross-sectional study

**DOI:** 10.1080/13814788.2017.1368490

**Published:** 2017-11-22

**Authors:** Barbara Wizner, Małgorzata Fedyk-Łukasik, Grzegorz Opolski, Tomasz Zdrojewski, Adam Windak, Marcin Czech, Jacek S. Dubiel, Michał Marchel, Krzysztof Rewiuk, Tomasz Rywik, Jerzy Korewicki, Tomasz Grodzicki

**Affiliations:** ^a^ Department of Internal Medicine and Gerontology, Jagiellonian University Medical College Krakow Poland; ^b^ Department of Cardiology, Medical University of Warsaw Warsaw Poland; ^c^ Department of Preventive Medicine and Education, Medical University of Gdansk Gdansk Poland; ^d^ Department of Family Medicine, Jagiellonian University Medical College Krakow Poland; ^e^ Department of Pharmacoeconomics, Medical University of Warsaw Warsaw Poland; ^f^ Business School, Warsaw University of Technology Warsaw Poland; ^g^ Department of Cardiology, Jagiellonian University Medical College and University Hospital Krakow Poland; ^h^ Department of Heart Failure and Transplantology, Institute of Cardiology Warsaw Poland

**Keywords:** Primary healthcare, chronic heart failure management, the elderly, cross-sectional study

## Abstract

**Background:** Organizational and educational activities in primary care in Poland have been introduced to improve the chronic heart failure (CHF) management.

**Objectives:** To assess the use of diagnostic procedures, pharmacotherapy and referrals of CHF in primary care in Poland.

**Methods:** The cross-sectional survey was conducted in 2013, involving 390 primary care centres randomly selected from a national database. Trained nurses contacted primary care physicians who retrospectively filled out the study questionnaires on the previous year’s CHF management in the last five patients who had recently visited their office. The data on diagnostic and treatment procedures were collected.

**Results:** The mean age ± SD of the 2006 patients was 72 ± 11 years, 45% were female, and 56% had left ventricular ejection fraction <50%. The percentage of the CHF patients diagnosed based on echocardiography was 67% and significantly increased during the last decade. Echocardiography was still less frequently performed in older patients (≥80 years) than in the younger ones (respectively 50% versus 72%, *Ρ* <0.001) and in women than in men (62% versus 71%, *P* <0.001). The percentage of the patients treated with β-blocker alone was 88%, but those with a combination of angiotensin inhibition 71%. The decade before, these percentages were 68% and 57%, respectively. Moreover, an age-related gap observed in the use of the above-mentioned therapy has disappeared.

**Conclusion:** The use of echocardiography in CHF diagnostics has significantly improved in primary care in Poland but a noticeable inequality in the geriatric patients and women remains. Most CHF patients received drug classes in accordance with guidelines.

KEY MESSAGES

- More attention is needed to include echocardiography in diagnostics of CHF in older patients, also due to a higher prevalence of chronic comorbidities associated with preserved ejection fraction.
- Most CHF patients received drug classes in accordance with guidelines, and no age/gender-related gap was observed in the neurohormonal blockade.

## Introduction

Chronic heart failure (CHF) affects about 26 million adults in the world [1], approximately 1–2% of the population [2], and projections show that its prevalence will increase further [3]. In 2012, CHF was the most prevalent cause of hospitalization among cardiovascular disorders in Poland and it was mainly related to a high number of early readmissions as it concerned up to 60% of CHF patients within 30 days of discharge [4,5]. Moreover, between 25% and 75% of these early re-hospitalizations may have been preventable if optimal management and more comprehensive care for CHF patients had been introduced [4,6,7]. During the last decade, most of the large studies conducted in Poland reported data on CHF management in hospital settings or cardiology outpatient clinics [8]. Meanwhile, some data indicate that Polish primary care physicians (family doctors and general internists) are key persons in taking care of CHF patients, and each primary care physician treats from 7–36 CHF patients [7]. These patients are usually older than CHF patients under the care of cardiologists [9].

Within the National Programme for Prevention of Cardiovascular Diseases (POLKARD 2005), a series of nationwide surveys was conducted in randomly selected primary care settings to assess adherence to guidelines on CHF management with regard to the diagnosis and treatment procedures [9–11]. The changes that have taken place in Poland over the last decade (see Box 1) have prompted a repetition of the survey in 2013. The aim of the present study was to assess the current CHF management in primary care settings in Poland. The study was designed to answer the following research questions:

1. Do Polish primary care physicians have access or perform basic diagnostic procedures for CHF patient?
2. What are the drugs prescribed to CHF patients by Polish primary care physicians?
3. What is the rate of referrals to the secondary care of CHF patients seen by primary care physicians?
4. Have diagnostic and therapeutic procedures for CHF patients changed since the previous survey dated in 2005?

## METHODS

### Study design

The study was a nationwide, retrospective survey performed according to the principles of the previous survey edition in 2005 [9,11]. A trained nurse contacted one physician drawn from the list of all doctors providing medical care at the selected primary care centre. The selected physician was asked to provide accurate data on the management of the last five CHF patients, consulted within the last 365 days before the survey. Data on patients’ characteristics (sex, age, NYHA class, and comorbidities), specific for CHF diagnostic procedures (echocardiography, NT-proBNP) and pharmacotherapy (class of drugs) have been collected. The field studies were carried out from April to November 2013.

### Selection of participating primary care centres

The sampling frame was the Polish nationwide registry of entities carrying out medical activities available at https://rpwdl.csioz.gov.pl—in April 2013. Out of all settings providing primary care a proportional stratified multistage sample of 390 primary care centres was selected. Random selection was held in each stratum defined by the province and size of the town in such a way that the number of primary care centres was proportional to the fraction of the population that the stratum constitutes in the general population. In the selected primary care centre, the physicians were randomly chosen from the list of primary care physicians employed in the centre.

### Inclusion and exclusion criteria

Patients who had ever been diagnosed with CHF were included in the study. The diagnosis of CHF had to be available in the patients’ medical records and the primary care physician had to be convinced of the presence of CHF (based on medical records at patients’ discharge from the hospital or a cardiologist) and treated that patient during the previous year as a CHF patient. CHF did not have to be the direct reason for encounter. A currently treated oncologic condition was a criterion for exclusion from the study.

### Statistical analysis

Continuous variables were summarized as mean ± standard deviation (SD). Ordinal variables or variables with skewed distribution were presented as median and interquartile range (IQR). Data on prevalence were given as numbers (percentages). The comparative analyses were based on Student’s *t*-test or the Wilcoxon test, and the chi-square test. The Cochrane–Armitage trend test was used to assess age-related changes in pharmacological therapy of CHF. Two-sided tests were used and the *P*-value <0.05 was considered as significant.

Data were managed and analysed using SAS v. 9.3 (SAS Institute Inc., Cary, NC, USA).

### Ethical issues

Data were collected and processed maintaining confidentiality and anonymity of the surveyed patients and the primary care physicians participating in the study. The study was approved by the Bioethics Committee of the Jagiellonian University (approval no. KBET/71/B/2011)—opinion dated 28 June 2012, and was valid until 31 December 2013.

## Results

The surveyed primary care centres mostly operated within the structures of private healthcare institutions, acting as an independent contractor within the public healthcare system. The participating physicians were mostly family physicians (63%) and specialists in internal medicine (38%), 7% were cardiologists and 2% were geriatricians.

The mean age (± SD) of the 2006 surveyed CHF patients was 72 years (± 11); 45% were women, 74% had ischaemic history of CHF. The baseline characteristics of the patients was summarized and compared from the study population from 2005 in Table 1. In comparison to the 2005 study, the surveyed patients were significantly older—the number of octogenarians increased by 10%. Moreover, slightly more CHF patients had a history of myocardial infarction, valvular heart disease and atrial fibrillation. A higher prevalence of chronic comorbidities was observed.

**Table 1.** Clinical characteristics of CHF patients in primary care settings in 2013 in comparison to 2005. Data are presented as means ± standard deviation or number (percentage).

	2013	2005	*P*-value
Number of patients’ medical records surveyed	2006	2000	
Women, *n* (%)	861 (44.8)	904 (47.0)	0.381
Age, years	71.9 ± 11.0	68.8 ± 11.8	
Age categories, *n* (%)			
<60 years	315 (15.7)	487 (24.3)	<0.001
60 − 69 years	414 (20.6)	433 (21.6)	
70 − 79 years	687 (34.3)	695 (34.8)	
80+ years	584 (29.1)	374 (18.8)	
Missing data	6 (0.3)	11 (0.5)	
NYHA class, *n* (%)			
I	41 (2.0)	79 (4.0)	<0.001
II	1117 (55.7)	936 (46.8)	
III	763 (38.0)	876 (43.8)	
IV	62 (3.1)	98 (4.9)	
Missing data	23 (1.2)	11 (0.5)	
Cardiovascular comorbidities, *n* (%)			
Hypertension	1646 (82.1)	1678 (83.9)	0.129
Coronary heart disease	1476 (73.6)	1632 (81.6)	<0.001
Myocardial infarction	748 (37.3)	682 (34.1)	0.035
Stroke or TIA	337 (16.8)	335 (16.8)	1.000
Dilated cardiomyopathy	312 (15.6)	349 (17.5)	0.106
Other cardiomyopathies	284 (14.2)	313 (15.7)	0.183
Valvular heart disease	583 (29.1)	402 (20.1)	<0.001
Atrial fibrillation	1010 (50.3)	768 (38.4)	<0.001
Other comorbidities, *n* (%)			
Diabetes	678 (33.8)	617 (30.9)	0.049
Chronic kidney disease	413 (20.6)	188 (9.4)	<0.001
Thyroid diseases	345 (17.2)	238 (12.0)	<0.001

CHF: chronic heart failure; NYHA: New York Heart Association; TIA: transient ischaemic attack.

### Diagnosis of heart failure

The diagnosis of CHF was supported by echocardiography in 67% of the patients in primary care settings surveyed in 2013, although the small changes regarding to the accessibility of the echocardiography in primary care settings were observed (Table 2). In general, diagnosis of CHF was based on the presence of CHF symptoms (93%) and results of ECG (82%) and/or chest X-ray (76%). Echocardiography was less frequently performed in women than in men (62% versus 71%, *P* <0.001) and in very old patients (≥80 years) than in younger subjects (50% versus 72%, *Ρ* <0.001). The data from the last echocardiography of the surveyed CHF patients, available for primary care physicians has shown the median of the left ventricular ejection fraction (LVEF) equal to 45% (interquartile range: 35–55%). Of these, 43% have the normal ejection fraction.

**Table 2.** Accessibility of echocardiography and NT-proBNP for the surveyed primary care centres and presentation of the available results. Data are presented as means ± standard deviation, median [upper - lower quartile] or number (percentage).

	2013	2005	*P*-value
Number of surveyed primary care centres	390	400	
*Accessibility of the diagnostic procedures:*			
Echocardiography, *n* (%)			
<1 month	53 (13.5)	52 (13.0)	<0.001
1–3 months	61 (15.6)	90 (22.5)	
>3 months	74 (19.1)	55 (13.8)	
Not available	176 (45.2)	166 (41.5)	
Missing data	26 (6.7)	37 (9.3)	
NT-proBNP, *n* (%)			
<1 month	77 (19.7)	14 (3.5)	<0.001
1–3 months	25 (6.5)	7 (1.8)	
>3 months	8 (2.1)	4 (1.0)	
Not available	232 (59.6)	311 (77.7)	
Missing data	48 (12.2)	64 (16.0)	
*Results available to primary care physicians*			
LVEF, %			
Available data, *n* (%)^a^	1342 (66.9)	683 (34.2)	<0.001
LVEF, mean ± SD	44.8 ± 13.1	45.4 ± 14.1	0.285
Reduced (<40%)	419 (31.2)	222 (32.5)	0.527
Borderline (40–49%)	347 (25.9)	161 (23.6)	
Preserved (≥50%)	576 (42.9)	300 (43.9)	
NT-proBNP, pg/ml			
Available data, *n* (%)^b^	79 (3.9)	–	
NT-proBNP, median [Q1–Q3]	1492 [850–3128]	n.a.	

NT-proBNP: N-terminal prohormone brain natriuretic peptide; LVEF: left ventricular ejection fraction; n.a.: not available.

^a^ The last available examination.

^b^ Measured within the last three months before the inclusion in the survey.

Access to the serum concentration of N-terminal of the prohormone BNP (NT-proBNP) has improved, although it was still infrequently used in primary healthcare and the procedure was made only in a small group of patients (Table 2).

### Other diagnostic procedures and specialist consultations

During the year preceding the survey 24-h ECG was performed in 24% patients, spirometry in 17%, an exercise stress test in 19%, and 4% of the patients had undergone ergospirometry or the six-minute walk test. In addition, 77% of the CHF patients were referred to cardiologists, however with advancing age, the frequency of the referrals steadily decreased—from 92% in patients below 50 years of age to 70% among octogenarians, and 60% in 90-year-old patients. To psychologists, physiotherapists or dieticians were referred respectively 10%, 8% and 4% of the patients. During the last visit, primary care physicians recognized functional disability, cognitive decline and depressive symptoms, in respectively: 31%, 21% and 8% of the patients.

### Pharmacotherapy

Usage of the drug classes dedicated to CHF therapy as well as other cardiovascular medications was summarized in Table 3.

**Table 3.** Pharmacological management of CHF and concomitant therapy of cardiovascular diseases in primary care settings – data from 2005 and 2013.

Pharmacotherapy in CHF patients^a^	2013	2005	*P*-value
Available data *n*	1384	2000	
Drugs used in the CHF patients, *n* (%)			
ACEIs or ARBs	1104 (79.8)	1641 (82.1)	0.093
sz-blockers	1213 (87.6)	1362 (68.1)	<0.001
ACEIs or ARBs and sz-blockers	982 (71.0)	1138 (56.9)	<0.001
Diuretics	1117 (80.7)	1490 (74.5)	<0.001
MRAs	577 (41.7)	963 (48.2)	<0.001
Digitalis	225 (16.3)	644 (32.2)	<0.001
Ivabradine	5 (0.4)	n.a.	
Other CV drugs, *n* (%)			
Lipid-lowering drugs	949 (68.6)	894 (44.7)	<0.001
Anti-arrhythmic drugs	87 (6.3)	107 (5.4)	0.270
Antiplatelet drugs	711 (51.4)	1004 (50.2)	0.492
Oral vitamin K antagonists	500 (36.1)	382 (19.1)	<0.001
NOACs	52 (3.8)	n.a.	
Potassium supplementation	422 (30.5)	505 (25.5)	0.001
Ca-channel blockers, dihydropyridine	345 (24.9)	368 (18.4)	<0.001
Ca-channel blockers non-dihydropyridine	14 (1.0)		
Alpha-blockers	120 (8.7)	105 (5.3)	<0.001
Trimetazidine	94 (6.8)	176 (8.8)	0.035
Nitrates	132 (9.5)	841 (42.1)	<0.001
Vasodilators (peripheral)^b^	132 (3.9)	n.a.	

CHF: chronic heart failure; ACEIs: angiotensin-converting enzyme inhibitors; ARBs: angiotensin II receptor blockers; MRAs: mineralocorticoid receptor antagonists; CV: cardiovascular; NOACs: non-vitamin K antagonist oral anticoagulants.

^a^ Data based on the medical records indicating drugs taken by the patients.

^b^ Vasodilators (peripheral): nicergoline, pentoxifylline.

There were no significant differences between women and men in the use of angiotensin inhibition therapy and β-blockers (alone or combined with angiotensin inhibition), as well as in MRAs and digitalis use. The only difference was in the use of diuretics—frequently more in women than men (85% versus 77%, respectively, *P* < 0.001).

A significant increase in the use of β-blockers alone or with angiotensin inhibitions were observed when compared to the previous survey from 2005 as well as the loss of discrepancies in the use of β-blockers in younger and older patients (Figure S1, available online). Moreover, the percentages of CHF patients on digitalis therapy were decreased, whereas with age a more frequent use of diuretics and less frequent use of MRAs were observed.

## Discussion

### Main findings

The study has indicated that in the two-thirds of the CHF patient’s diagnosis has been confirmed by echocardiography, more often in the younger patients and men. The use of natriuretic peptides measurements in diagnostics of CHF was insufficient in everyday medical practice. Most of the CHF patients have received the drug classes according to the guidelines but there is still room for improvement. Compared to the previously study edition, the percentage of CHF patients in primary care whose diagnosis was supported by echocardiography significantly increased. Regarding the CHF therapy, a significant increase in the usage of neurohormonal inhibitors was observed, especially in the patients over 70 years of age.

### Interpretation of the study—the results in relation to existing literature

Observed in our survey, a better implementation of echocardiography in primary care in Poland suggests that CHF diagnostics is more objective and more compliant to the current ESC guidelines. In our study, 67% of the CHF patients had undergone echocardiography and these results were available for primary care physicians. Very similar data were obtained in an Italian study, where 57% of all subjects had a diagnostic echocardiography record [12]. Moreover, the age-related inequalities observed in our study were also visible in the above-mentioned study as well as in other countries, both in primary care and in long-term care settings [13,14].

The data on CHF diagnosis as well as the management obtained from primary care settings are more difficult to evaluate because of the frequent problem with validation of CHF diagnosis. Some studies highlighted the problem of either misdiagnosing or failing to recognize CHF as well as overdiagnosis of CHF in primary care [14–18]. In Poland, echocardiography is still not a diagnostic procedure routinely available in primary care settings; therefore, the effort should be probably provided to more common use of natriuretic peptides diagnostic tests [6,7]. Taking into account the recently published data [16], some indirect evidence for a truly existing CHF in our patients may be history of cardiologic consultation—77% of the surveyed patients were consulted during the last year by the cardiologist. In addition, the reliability of the CHF diagnosis increase the ischaemic aetiology of CHF observed in most of our patients.

The study findings confirm that noticeably more CHF patients in primary care settings are currently being treated in line with evidence-based medicine and the current recommendations [6,7,19]. In Poland, 15 years ago, ACEIs, β-blockers, diuretics and digitalis were used by 65%, 34%, 57% and 39% of CHF patients [20].

Comparing our findings to the data from the ESC-HF registry, originating from a similar period, we found that the use of β-blockers, diuretics and ACEIs was similar despite differing settings of care and was 89%, 83% and 67%, respectively [8]. Reduced use of MRAs in our survey (42% versus 59%) can be explained by the significantly older CHF population—more than 60% of our patients were 75 years of age and older, compared to 26% of the patients in the ESC-HF registry [8]. Moreover, approximately 50% of our subjects had a normal LVEF—this is generally in line with the data on the prevalence of CHF with preserved LVEF [7,21]. In light of the existing evidence, the analysis of guideline adherence for the pharmacotherapy of CHF in our population should in fact, be limited to using the neurohormonal blockade as an effective method in the reduction of all-cause mortality in CHF with preserved LVEF [22,23].

As CHF patients in primary care settings are usually older [9,18] and suffer from multimorbidity [24], for optimal management, it appears necessary to look at them through the prism of a comprehensive geriatric assessment [19]. The published studies confirm that functional decline, frailty syndrome, depression and cognitive impairment are independent predictors of short- and long-term mortality among elderly patients with CHF [25–27].

### The strength and limitations of the study

The strength of the study is the large number of the surveyed primary care centres and nationwide coverage. Our study seems to reflect everyday practices in the management of CHF patients in primary care because the data comes from patients who have already been diagnosed and are currently treated due to CHF.

One of the main limitations of the present study is the potential selection bias of the patients reported by primary care physicians. Despite strong recommendations for the physicians and detailed information provided by specially trained nurses that CHF patients should be recruited as the last five to have recently visited the office of a given primary care physician, we cannot rule out the possibility that the selection of patients was based on the primary care physicians’ preferences.

Due to the retrospective study design, a major limitation of our study seems to be the lack of external verification of the CHF diagnosis as well other collected data.

These study limitations occurred both in the 2005 and 2013 surveys.

An additional limitation in 2013 was a high percentage of missing data in echocardiography results, external consultations as well as pharmacotherapy regimen partly resulting from the gaps in the patients’ files. Because of the inability to verify the data on pharmacotherapy, we decided to exclude such incomplete or uncertain data from our analysis.

### Recommendations for clinical practice and further research

Keeping in mind that in Poland echocardiography is a diagnostic procedure, which is not performed directly in primary care settings, the results of the study should open the discussion how to change the situation and improve the accessibility, both to echocardiography and NT-proBNP serum concentration for the patients of a family physician. Assessment of the effectiveness of their use in primary care settings should be the subject of future research. A further popularization of CHF guidelines is needed to optimize the pharmacotherapy regimen in CHF patients, both in regard to the use of recommended class of drugs (e.g. angiotensin inhibition) and probably the optimal doses of drugs. Given that the CHF population is getting older, a further educational effort is needed to improve the implementation of geriatric evaluation basis to the clinical practice of family physicians.

## Conclusion

During the last decade, an improvement in the use of echocardiography in CHF diagnostics in primary care has been observed in Poland, although its accessibility has not improved. A minority of the primary care centres declared access to NT-proBNP measurement and only a few percent of the CHF patients had this diagnostic test performed. There still exists an inequity in performing echocardiography concerning geriatric patients and women. Such disparities were not visible in the first-line CHF treatment drug classes. Most CHF patients received drug classes in accordance with guidelines.

## Supplementary Material

Figure S1
