# Supplementary material for: Chronic heart failure management in primary healthcare in Poland: Results of a nationwide cross-sectional study
Source: Eur J Gen Pract. 2017 Nov 22;24(1):1–8. doi: 10.1080/13814788.2017.1368490 (PMC5774260; doi:10.1080/13814788.2017.1368490)

Figure S1. Pharmacological management of CHF patients in PC settings regarding their age – data from 2013 in comparison to 2005.

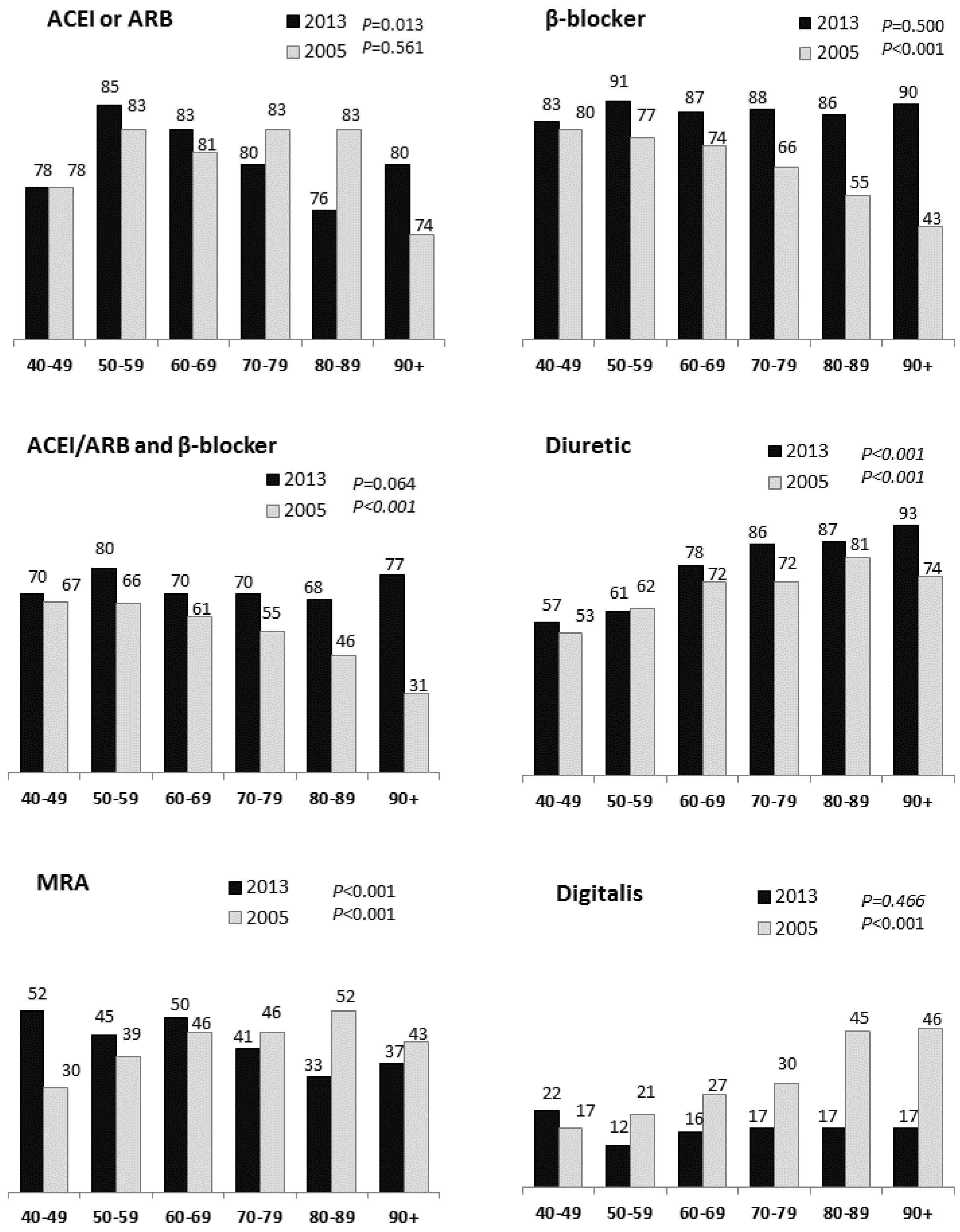

Supplement: Figure S1 [file IGEN_A_1368490_SM9782.pdf]
